# A Computational Study of Calcium(II) and Copper(II) Ion Binding to the Hyaluronate Molecule

**DOI:** 10.3390/ijms130912036

**Published:** 2012-09-20

**Authors:** Elizabeta Tratar Pirc, Jernej Zidar, Peter Bukovec

**Affiliations:** 1 EN-FIST Centre of Excellence, Dunajska 156, Ljubljana SI-1000, Slovenia; E-Mail: peter.bukovec@fkkt.uni-lj.si; 2 Faculty of Chemistry and Chemical Technology, Aškerčeva 5, Ljubljana SI-1000, Slovenia; 3 Institute of High Performance Computing, Agency for Science, Technology and Research, 1 Fusionopolis Way, #16-16 Connexis North, SG-138632, Singapore; 4 National Institute of Chemistry, Hajdrihova 19, Ljubljana SI-1000, Slovenia

**Keywords:** copper(II) ion, calcium(II) ion, hyaluronic acid, QM/MM, simulation, biopolymer

## Abstract

The hyaluronate molecule is a negatively charged polysaccharide that performs a plethora of physiological functions in many cell tissues depending on its conformation. In the present paper, molecular modeling at three levels of theory and two basis sets was used to gain a deeper insight in the complex molecular structure of calcium(II) and copper(II) hyaluronate. Simulation results were compared with the experimental data (EXAFS or X-ray). It was found that B3LYP does not properly reproduce the experimental data while the HF and M06 methods do. Simulation data confirm that the *N*-acetyl group of the *N*-acetylglucosamine residue does not participate in the coordination bonding to the calcium(II) or copper(II) ion, as evident from the experimental data.

## 1. Introduction

Hyaluronic acid (HA) is a high molecular weight polysaccharide present in the extracellular matrix of most vertebrate tissues [1]. Its functions vary from maintaining the constant volume of the interstitial fluid, to organizing the extracellular matrix and various immunosuppressive functions [2]. The presence of HA on plasma membranes and the variation of its concentration in the pericellular spaces is associated with cell aggregation during morphogenesis and formation of metastasis during malignant transformation and invasion of tumors [2–5].

HA is a polymer composed of linear repeats of the disaccharide unit containing d-glucuronic acid (GCU) and *N*-acetyl-d-glucosamine (NAG). A typical hyaluronate molecule can consist of as many as 10.000 disaccharide units. The overall negative charge of a HA molecule under physiological conditions is caused by the repeating disaccharide units containing anionic carboxylic sites [3]. The interaction of those sites with metal cations is an important factor contributing to the overall supermolecular structure of HA [6]. Other factors include: the type of counter ion, pH, temperature and the extent of hydration; with the type of counter ion being the most important [7,8]. Structural and literature data for transition metal complexes with HA remain scarce and limited to coordination complexes in water solution with Ca^2+^, Ag^1+^, Cd^2+^, Pb^2+^, Fe^3+^ [7,9–11].

In the past, X-ray fiber diffraction was successfully used to solve the solid state structure of HA containing various cations from the 1st and 2nd group of the periodic table, where the formation of 2- or 4-fold helices was reported [12–15]. The conformation of the polyanion is stabilized by hydrogen bonds across the glycosidic linkages between the HA monomers. Adjacent antiparallel chains are held together through –COO^−^–Ca^2+^–^−^OOC– bridges and six hydrogen bonded water molecules [14]. It has been suggested, that the secondary structure of the polymer will be similar to calcium(II) hyaluronate also in cases of other divalent cations [15].

Amorphous bivalent metal hyaluronates containing either copper(II), or nickel(II), or manganese(II) or cobalt(II) ions were prepared at pH 5.5 by precipitation from water solutions with cold ethanol. The local structure around the metal(II) ion was determined by EXAFS and XANES method [16,17]. The coordination polyhedron around the copper(II) ion was shown to be a distorted octahedron. Four oxygen atoms at an average distance of 1.95 Å were found to occupy the planar equatorial sites. At the axial sites oxygen atoms are present at 2.46 Å. Though oxygen atoms are preferred at the axial positions, nitrogen atoms from the *N*-acetyglucosamine residue cannot be excluded as well [16].

Using quantum chemical methods the basic disaccharide unit of HA was studied. Semiempirical MO methods and *ab initio* calculations have shown good agreement between the optimized geometries and available crystallographic data [6,18].

As reported in [16,17], synthesized calcium and copper(II) hyaluronates used in the study are amorphous materials making the analysis difficult as X-ray diffraction patterns cannot be used to explain the experimental EXAFS data. One possible way is to use a combined quantum mechanical (QM)/molecular mechanics approach (MM) [19]. This approach was successfully used in computational enzymology, it was also employed in studies of metal ion binding to a protein and HA as well [20–22]. Transition metal binding was also studied using DFT methods [23–27]. In all the cases mentioned, atomistic simulations proved to be an invaluable tool for elucidation of experimental structural data.

Larger systems can be studied using a combined quantum mechanical (QM)/molecular mechanics description (MM) [19]. While this approach was successfully used in computational enzymology, it was also employed in studies of metal ion binding to a protein and HA as well [28–33]. In the latter case, QM/MM proved to be an invaluable tool for elucidation of experimental structural data.

In this article we performed QM/MM calculation of complexation of hyaluronate molecule with either calcium(II) or copper(II) ions. We considered Hartree-Fock, B3LYP and M06 methods in conjunction with flexible basis sets, while the part of the system described on the molecular mechanics level was described by the CHARMM force field [23–28]. Obtained geometric parameters were compared with experimental values from X-ray diffraction for calcium(II) hyaluronate and EXAFS for copper(II) hyaluronate. We demonstrated that the M06 functional proposed by Zhao and Truhlar and co-workers adequately describes the geometric properties of both calcium(II) and copper(II) hyaluronate system [30].

## 2. Results and Discussion

**Figure 1:**
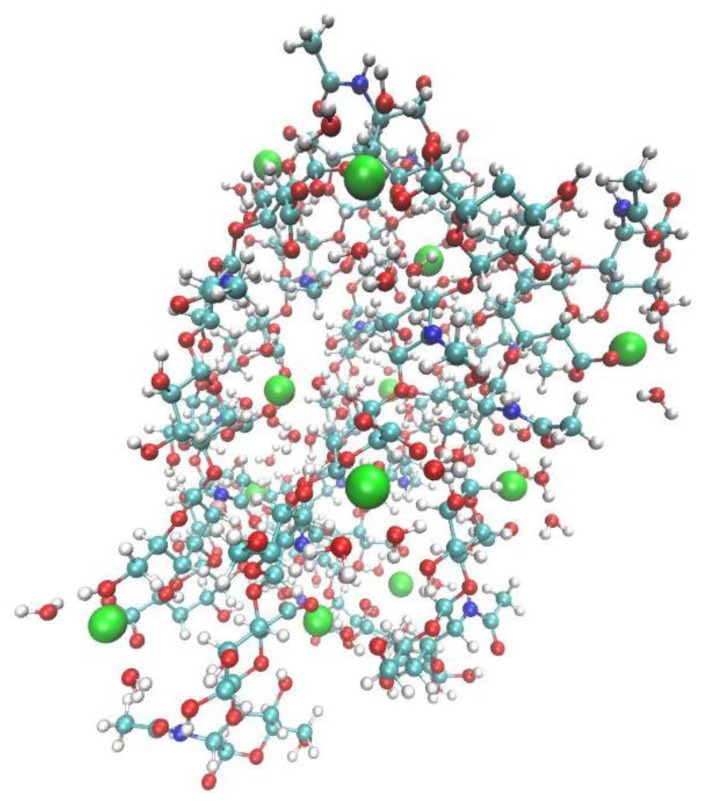
Model hyaluronate unit. Calcium(II) ions are depicted as Van der Waals spheres. Key to atom colours: white-hydrogen, red-oxygen, cyan-carbon, blue-nitrogen, green-calcium(II) ion.

The starting system was constructed from PDB data taking into account the fact the biologically active form is a hexamer [14,15]. The initial structure of calcium(II) hyaluronate prepared from PDB data is displayed in Figure 1, details are available elsewhere [22].

The final distances between the central calcium(II) ion in the QM region and the ligands after the minimization are collected in Table 1. Our data show the best agreement with experimental X-ray data is obtained if the system is minimized using the M06 method and the basis set 6-31G(d,p), while using HF or B3LYP does not. Since the calcium ion is a rather large cation (radius 0.99 Å) the coordination numbers are between 6 and 8. In calcium hyaluronate it accommodates eight ligands, in this case the carboxylate groups of two glucuronic acid residues and six neighboring water molecules.

In the next step the calculated distances between the ligand and central atom from Table 1 were processed in order to obtain the data presented in Table 2 (see section Methods for details). The processed data for calculation for calcium hyaluronate indicate the standard deviation is the lowest if the simulation is performed with M06 and the basis set 6-31G(d,p). Since the M06 method was parameterized with transition metals and non-metals, this indicates M06 adequately describes the coordination behavior of calcium(II) ion in the hyaluronate complex.

By replacing the calcium(II) ion with copper(II) we obtain a new system. Though the two ions (calcium(II) and copper(II)) have the same charge, their behavior is different.

Table 3 summarizes the distances between the central ion and the ligands in the first coordination shell after the QM/MM minimizations using HF, B3LYP or M06 and the 6-31G* or 6-31G(d,p) basis set. EXAFS experimental data showed that the copper(II) ion is coordinated by four equatorial oxygen atoms at an average distance of 1.95 Å. For the two axial sites at an average distance of 2.46 Å, oxygen atoms were preferentially indicated, although the participation of nitrogen atom from the *N*-acetylglucosamine residue could not be excluded as well. The coordination polyhedron of the copper(II) ion is therefore a disordered octahedron, due to the Jahn-Teller effect [16]. This so-called tetragonal distortion is the consequence of the *d*^9^ configuration since the *t*_2g_ level is not symmetrically filled with electrons (three electrons in two orbitals).

**Figure 2:**
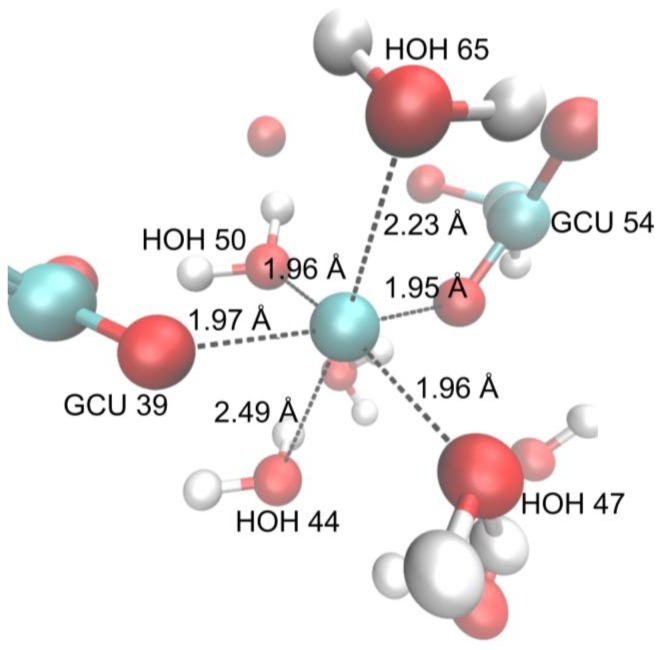
Copper(II) hyaluronate complex after minimization on M06 level with basis set 6-31G* with distances to copper(II) ion indicated.

The final structure of copper(II) hyaluronate after the QM/MM minimization using M06 and the basis set 6-31G* confirms the EXAFS data (see Figure 2). Four oxygen atoms are present in the equatorial plane at approximately 1.96 Å. Two oxygen atoms come from two water molecules, whereas two oxygen atoms come from the carboxylate groups of the nearby GCU residues. The axial oxygen atoms come from two water molecules. Our results indicate the nitrogen atom from the *N*-acetylglucosamine residue does not participate in the coordination bonding as it is located outside the first coordination sphere.

The radius of the copper(II) ion is smaller (*i.e.*, 0.73 Å) than the radius of the calcium(II) ion (0.99 Å) therefore a different binding behaviour was expected. The electronic configuration of the copper(II) ion is *d^9^*, with three electrons occupying the orbitals *d*_x2–y2_ and *d*_z2_. Since the *t*_2g_ level is asymmetrically occupied (three electrons in two orbitals), *e*_g_ and *t*_2g_ orbitals are no longer degenerated, resulting in unequal distances between the central atom and the ligands at the axial and equatorial positions. The results in Table 3 show the aforementioned differences. HF and M06 are in good agreement with experimental data obtained by EXAFS regardless of the basis set used. During the B3LYP simulations we observed two water molecules were pulled outside of the first coordination shell.

The data in Table 3 were then processed in the same manner as the data in Table 1. The calculated standard deviations presented in Table 4 indicate once more that the best agreement with experimental data is obtained with the M06 method regardless of the basis set used; the second best agreement is obtained when using HF.

For more than a decade B3LYP was the functional of choice for studies of many organic systems as evident by the many references in literature [23–27]. The case of calcium(II) and copper(II) ion binding to hyaluronic acid is one, where the results (*i.e.*, computed distances between the central atom and the ligands) given by the B3LYP functional greatly differ from the experimental values obtained by either EXAFS (for copper(II) hyaluronate) or X-ray (calcium hyaluronate). One reason may lay in the difficulty in describing the nonbonding interactions between the central atom and the ligands that M06 seem to adequately account for.

Our minimizations of calcium hyaluronate began from the experimental structure. After the minimization using either Hartree Fock or M06 but not B3LYP, we were able to replicate the experimental values. This indicates that both the starting model and the methods employed adequately describe the nonbonding interactions between the central atom and the ligands for both the cases evaluated in this study. As in almost all the systems of biological interest, electrostatics is the main component of the nonbonding energy. In the complexes we studied the ionic nature of the binding metals makes it even more important. Dispersion component of the interaction energy is far less relevant for our systems. Again, quantum chemical calculations include all the components of the interaction energy and the applied M06 functional properly accounts for the dispersion component.

One possible explanation for this is the fact that we were trying to mimic the environment within the solid state calcium and copper(II) hyaluronates for which we had experimental data. Within the solid state the conformational space is severely limited which in turn means that regardless of the starting point (*i.e.*, either starting with calcium or copper(II) ion) the system will end up in the global minimum. Also, since the conformational space within the crystal seems to be relatively small the problem of several local minima and there with associated need for thermal averaging is to a large extent circumvented.

## 3. Methods

The initial calcium(II) hyaluronate model was constructed as described previously [22] with the main difference being the size of the QM region as advances in computer hardware allowed us to define a larger QM region, which substantially improves the reliability of results.

The QM region consisted of: a calcium(II) ion, two carboxylate groups from two GCU residues (labelled GCU 39 and GCU 54, respectively), two *N*-acetyl groups from two NAG residues (labelled NAG 38 and NAG 53, respectively) and six water molecules (labelled HOH 44, HOH 47, HOH 50, HOH 59, HOH 62, HOH 65). To delimit the QM region from the MM region dummy link atoms were used on residues NAG 38, GCU 39, NAG 53 and GCU 54.

The QM region was simulated with GAMESS-US using either Hartee-Fock or B3LYP or M06 and using either the basis set 6-31G* and 6-31G(d,p) [29]. The rest of the system was simulated by molecular mechanics with the CHARMM force field, a constant dielectricity of 1.0 and a cut-off distance of 12.0 Å. All simulations were carried out at 300 K. The minimizations were run for 1000 steps. The trajectories were analyzed and the results were compared to available X-ray data for calcium(II) hyaluranate and EXAFS data for copper(II) hyaluronate [15,16].

Previous experimental data show the overall conformation of the hyaluronic acid does not depend much on the bound ionic species, thus calcium(II) ions can be replaced with copper(II) ions. Calculations were subsequently reran and the results were compared with experimental X-ray data for calcium(II) hyaluronate and EXAFS for copper(II) hyaluronate [15,16].

The computed distances at the end of the minimization were the used to calculate the quotients between the computed distances for a given central atom-ligand pair with the experimental distance from the same pair, afterwards the standard deviation was computed for each combination of method and basis set. The values are collected in Table 2 for calcium hyaluronate and Table 4 for copper(II) hyaluronate.

For the QM/MM simulations the molecular modeling package CHARMM with the GAMESS-US was used, version c35a2q, and ran on the CROW computer clusters at National Institute of Chemistry in Ljubljana [32–35]. Molecular graphics were produced using VMD [36].

## 4. Conclusions

A series of QM/MM minimizations of calcium(II) hyaluronate and copper(II) hyaluronate revealed differences in the distribution of ligands around the central ion in the first coordination shell. Simulations were performed using Hartree-Fock, B3LYP, M06 and two different basis sets. The simulated distances between the ligands and the central ion at M06 level are in good agreement with experimental data for both, calcium(II) and copper(II) hyaluronate, respectively.

There are two main characteristics of Ca(II) and Cu(II) ions to be considered when comparing the environment of both cations with the same ligand. The first characteristic is the size of the cation, and the second one is its electronic configuration. They both influence the coordination behavior in a specific way. For cations such as Ca(II) with symmetrical charge distribution (empty d orbitals) there is no preferential stereochemical direction to be favored by the cation. The size of Ca(II) ion plays the main role in defining its coordination number, at least in simple complexes. It is well known that coordination numbers 6 and 8 are favored for Ca(II). In case of calcium hyaluronate the coordination number is 8, which was determined using X-ray diffraction and confirmed by molecular modeling.

On the other hand, Cu(II) ion is much smaller, and on that basis the preferred coordination numbers are 4 and 6. The *d*^9^ configuration makes Cu(II) subject to Jahn-Teller distortion if placed in an environment of cubic symmetry, and this has a profound effect on all its stereochemistry. When the coordination number is six, the octahedron is severely distorted with an elongation along one fourfold axis as a typical distortion. Our findings for copper(II) hyaluronate confirm such behavior.

Large multidentate ligands like hyaluronic acid do not obey the simple size-ratio rule. The steric flexibility of the ligand to approach its donor atoms against the central cation may be the prevalent factor in complex formation. It was observed that the local conformation of the hyaluronate chains depends more on the charge and size of the cation and less on its type [12]. Also both experimental and our simulation data indicate the nitrogen atom from the *N*-acetylglucosamine residue does not participate in the coordination bonding to either calcium(II) or copper(II) ions. Within the solid state both our system mimic the conformational space is severely limited which in turn means that regardless of the starting point (*i.e.*, either starting with calcium or copper(II) ion) the system will always end up in the global minimum after the minimization. Our preliminary simulations of other transition metal hyaluronate complexes further confirm this finding.

## Figures and Tables

**Table 1:** Distances between the calcium(II) ion and the ligands after the minimization using HF, B3LYP, M06 and two basis sets (6-31G* and 6-31G(d,p), respectively). In the second column experimental X-ray data are reported [14].

Ligand	EXP [Å]	HF [Å]		B3LYP [Å]		M06 [Å]	
		
		6-31G *	6-31G(d,p)	6-31G *	6-31G(d,p)	6-31G*	6-31G(d,p)
GCU 39 O6A	2.510	2.408	2.416	2.408	2.469	2.554	2.384
HOH 44 O	2.474	2.495	2.522	2.468	2.479	2.587	2.498
HOH 47 O	2.556	2.456	2.505	2.421	2.482	2.598	2.558
HOH 50 O	2.569	2.418	2.415	2.401	2.376	2.278	2.395
GCU 54 O6A	2.511	2.412	2.434	2.407	2.470	2.578	2.418
HOH 59 O	2.475	2.518	2.553	2.440	2.532	2.604	2.507
HOH 62 O	2.555	2.494	2.525	2.463	2.509	2.250	2.581
HOH 65 O	2.570	3.444	3.632	3.327	3.639	2.599	2.637

**Table 2:** Standard deviation of the quotient of the ligand-central atom distance for calcium(II) hyaluronate calculated using the experimental (2nd column) and simulation data.

	EXP	HF		B3LYP		M06	
			
Deviation		6-31G*	6-31G(d,p)	6-31G*	6-31G(d,p)	6-31G*	6-31G(d,p)
	1.000	0.132	0.154	0.112	0.156	0.068	0.035

**Table 3:** Distances between the copper(II) ion and the ligands after the minimization using HF, B3LYP, M06 and two basis sets (6-31G* and 6-31G(d,p), respectively). In the second column experimental EXAFS data are reported [16].

Ligand	EXP [Å]	HF [Å]		B3LYP [Å]		M06 [Å]	
		6-31G *	6-31G(d,p)	6-31G *	6-31G(d,p)	6-31G *	6-31G(d,p)
GCU 39 O6A	1.952	1.976	1.983	1.917	1.969	1.966	1.989
HOH 44 O	2.465	2.341	2.371	3.458	2.901	2.487	2.385
HOH 47 O	1.952	2.070	2.072	1.967	1.990	1.960	2.002
HOH 50 O	1.952	2.070	2.069	2.032	1.998	1.960	1.997
GCU 54 O6A	1.952	1.992	2.002	1.946	2.003	1.954	1.982
HOH 59 O	3.767	3.829	3.892	3.904	3.705	3.576	3.696
HOH 62 O	3.507	3.639	3.609	3.281	3.526	3.503	3.487
HOH 65 O	2.465	2.278	2.319	2.184	2.309	2.235	2.291

**Table 4:** Standard deviation of the quotient of the ligand-central atom distance for copper(II) hyaluronate calculated using the experimental (2nd column) and simulation data.

	EXP	HF		B3LYP		M06	
			
Deviation		6-31G *	6-31G(d,p)	6-31G *	6-31G(d,p)	6-31G *	6-31G(d,p)
	1.000	0.049	0.043	0.157	0.069	0.037	0.034
